# Towards a deeper haplotype mining of complex traits in rice with RFGB v2.0

**DOI:** 10.1111/pbi.13215

**Published:** 2019-07-23

**Authors:** Wang Chun-Chao, Hong Yu, Ji Huang, Wen-Sheng Wang, Muhiuddin Faruquee, Fan Zhang, Xiu-Qin Zhao, Bin-Ying Fu, Kai Chen, Hong-Liang Zhang, Shuai-Shuai Tai, Chaochun Wei, Kenneth L. McNally, Nickolai Alexandrov, Xiu-Ying Gao, Jiayang Li, Zhi-Kang Li, Jian-Long Xu, Tian-Qing Zheng

**Affiliations:** 1 Institute of Crop Sciences/National Key Facility for Crop Gene Resources and Genetic Improvement, Chinese Academy of Agricultural Sciences, Beijing, China; 2 State Key Laboratory of Plant Genomics and National Center for Plant Gene Research (Beijing), Institute of Genetics and Developmental Biology, Chinese Academy of Sciences, Beijing, China; 3 National Key Lab of Crop Genetics and Germplasm Enhancement, College of Agriculture, Nanjing Agricultural University, Nanjing, China; 4 Shenzhen Institute of Breeding for Innovation, Chinese Academy of Agricultural Sciences, Shenzhen, China; 5 Beijing Key Laboratory of Crop Genetic Improvement / College of Agronomy and Biotechnology, China Agricultural University, Beijing, China; 6 BGI Genomics, BGI-Shenzhen, Shenzhen, China; 7 Department of Bioinformatics and Biostatistics, School of Life Sciences and Biotechnology, Shanghai Jiao Tong University, Shanghai, China; 8 International Rice Research Institute, Metro Manila, The Philippines; 9 University of Chinese Academy of Sciences, Beijing, China

**Keywords:** phenotype-haplotype real-time assciation, webservice, deep haplotype mining, rice (*Oryza sativa* L.)

Rice (*Oryza sativa* L.) not only provides insurance covering global food security but also works as a model for plant research. Currently, with overwhelmingly accumulated sequencing data, various databases were constructed for different target users, including the Genome Variation Map (Song *et al*., 2018), RiceVarMap (Zhao *et al*., 2015), SNP-Seek database (Alexandrov *et al*., 2015), RPAN (Sun *et al*., 2017) and MBK V1 (Institute of Genetics and Developmental Biology, C.A.S., 2018). However, none of them is designed to meet increasing demands of correlation mining between huge sets of phenotypic and genotypic data of re-sequenced genome. Online tool for deeper mining of favourable allele/haplotypes is in urgent need.

In 2015, prototype of Rice Functional and Genomic Breeding (RFGB) was developed for breeding application (Zheng *et al*., 2015) based on SNP & InDel data from the 3000 rice genome (3K-RG) project (The-3K-rice-genomes-project, 2014). In order to bridge huge gaps between phenotypic and genotypic data sets of sequenced genome, our newly designed web service named RFGB v2.0 is now formally online (http://www.rmbreeding.cn/Index/).

RFGB v2.0 contains five major modules, including Phenotype, Haplotype, SNP & InDel, Germplasm and Restore Sequence. The phenotypic data for 3K accession were embedded in the Phenotype module. Currently, data of 12 traits are already publicly accessible. To improve the adaptivity, a function of real-time analysis with user uploaded phenotypic data is provided. In the analysis function embedded in the Haplotype module, non-synonym SNPs were considered. The functions of allelic frequency (MAF) screening and sample definition are also available. In the SNP & InDel module, information of a gene query is accessible by searching with either locus ID, chromosome region or even key words. In the Germplasm module, grouping of the 3K germ-plasms has been updated according to our recent report (Wang *et al*., 2018). The germplasm data set was re-organized to improve data efficiency. Additionally, we have corrected calculation errors in the trial version of the Restore Sequence module with full consideration of InDel. More details about the RFGB v2.0 construction and utility methodologies are accessible through our online materials (http://www.rmbreeding.cn/Index/manual).

Four typical user cases picked from our web tests were presented here: 1) exploring favourable donors, 2) shortlisting candidate genes, 3) mining favourable haplotypes and 4) seeking variations and restore sequences.

**Figure 1:**
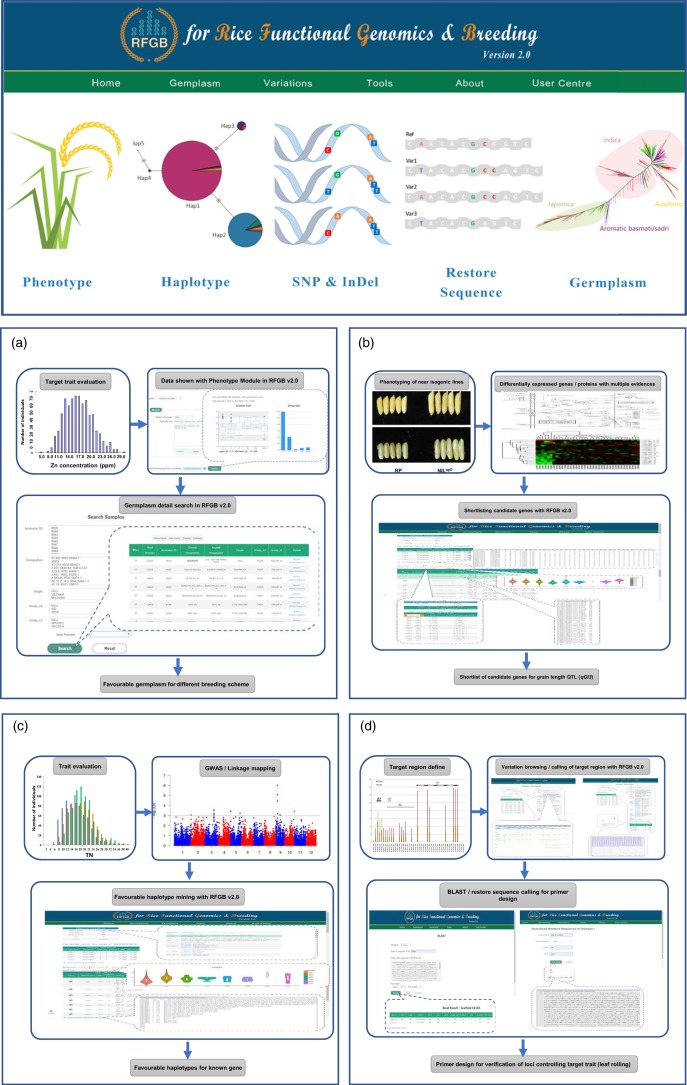
Four user cases working on exploring favourable donors, shortlisting candidate genes, mining favourable haplotypes and seeking target sequence/variations using RFGBv2.0. (a) User analysed phenotypic distribution by uploading zinc concentration in milled grains (Zn) data using the Phenotypic module. With the information of elite group for Zn, more details of favourable germplasms for different breeding schemes were accessible using the Germplasm module. (b) With aid of the Haplotype module, a list of 22 differentially expressed genes (DEGs) based on the transcriptomic and phosphoproteomic analyses between near-isogenic line (NIL^*qgl3*^) and its recurrent parent (RP) were significantly reduced by 31.8%. (c) Based on GWAS mapping results of tiller number (TN) in a set of germplasms, some candidate regions were submitted to the Haplotype module for confirmation by haplotype analysis. (d) A chromosome region controlling leaf rolling trait (LRI), *qRl4-2* was scanned for SNP & InDel variations using the SNP & InDel module. With the Restore Sequence module, restore sequence information was available for design of primers to confirm the LRI candidate region.

A typical pre-breeding/forward genetics work begins with phenotyping. As shown in Figure 1a, we adopted our published data set of zinc concentration in milled grains (Zn) (Zhang *et al*., 2018) with normal distribution as a sample case. Interestingly, by uploading data to the Phenotype module, user found that Zn, the custom trait (uploaded data), varied between different groups of the 3K-RG with the box-plot viewer. Three groups of the 3K-RG germplasms were found to present top Zn values, which are *Geng/japonica* (GJ), Aus and Basmati (Bas) groups. For breeding schemes working on *Geng/japonica* cultivar development, germplasm from GJ group with higher Zn values would be recommended as breeding donors. On the contrary, for a *Xian/indica* breeding scheme, donors from Aus and/or Bas group would be favourable. Further details for the above donors are accessible using the Germplasm module.

As shown in Figure 1b, a route for shortlisting candidate genes with RFGB v2.0 was supported by a user case working on grain length (GL) dissection with four steps: 1) construct near-isogenic lines (NIL) for the target locus; 2) set up multiple omics analysis for the NIL and recurrent parent (RP) to get a list of differentially expressed genes (DEGs); 3) use the Haplotype module for haplotype–phenotype association analysis; and 4) shortlist the DEG list based on comparisons of the target trait differences between haplotypes. In the GL case, transcriptomic and phosphoproteomic analyses were carried out for NIL^*qgl3*^ and RP, which were found to be significantly different in GL. A total of 22 DEGs were found. Then, we carried out haplotype analysis with the GL data set embedded in RFGB v2.0, which has more than 2,000 valid GL data points at present. With the parameter setting of MAF = non-filtered, the candidate gene list was shortened by dropping 31.8% genes off the list which were found to be non-related GL with intuitive supporting evidences from the Haplotype module.

As shown in Figure 1c, a route for mining favourable haplotypes with RFGB v2.0 includes four major steps: 1) trait evaluation for the germplasms from the 3K-RG; 2) definition of candidate regions by GWAS/linkage mapping; 3) shortlist candidate genes with multiple evidences; and 4) explore favourable haplotypes of candidate genes for target traits. In this case, by GWAS mapping, we found a loci *qSV3e*, which was harbouring a known gene Os03 g0856700 coding Gibberellin 20 oxidase 1 affecting both tiller number (TN) and plant height (PH) at the seedling stage under a paddy direct seeding rice (PDSR) system. Since relatively higher TN would be favourable trait for rice breeders, we carried our haplotype analysis for Os03 g0856700 with TN data uploaded using the Haplotype module. We found that Hap 7 of Os03 g0856700 contributed more to TN than the other haplotypes and could be adopted as candidate elite haplotypes for rice molecular breeding under PDSR system. A donor list of 16 3K-RG germplasms carrying Hap 7 were also accessible in the Haplotype module.

In addition to the above explorations, a route for variation mining with RFGB v2.0 includes the following three steps (Figure 1d): 1) define a target region for targeting trait with partial accessions from the 3K-RG, 2) access more variations within the target region with the SNP & InDel module and 3) access the restore sequences harbouring the targeting variations using the Restore Sequence module for further wet-lab confirmation. In our user case, a target region, *qRl4-2*, was defined with GWAS with no more than 1000 accessions for a targeting trait, leaf rolling index (LRI). With the JBrowse engine embedded in the SNP & InDel module, a deeper mining of variations within *qRl4-2* in more than 2000 additional germplasm was feasible. Restore sequences of elite germplasms were then downloaded according to the sub-region harbouring the genome variations according to genome browsing with the SNP & InDel module. Further wet-lab work would be carried out based on these results.

RFGB v2.0 has offered a unique view on the relationships between two big data sets (phenotypic and genotypic data) of sequenced genome, especially a real-time analysis for the phenotype–haplotype associations. With different combinations of the modules and functions embedded in RFGB v2.0, users may feel free to have a deeper view of targeting complex trait. This may inspire more ideas on deeper mining of complex traits with sequenced genome data. Finally, in order set up an open platform to the public, two functions are now available. One is ‘Seed request’ (http://www.rmbreeding.cn/public/request_germplasm) which helps users to access the 3K-RG seeds more easily for their own phenotyping works. The other is ‘contribute your data to RFGB’ (http://www.rmbreeding.cn/Phenotype/contribute_phenotype) which helps users upload their own phenotypic data to RFGB. With respect to intellectual property, at present, only the published data are fully open for download.
